# Tertiary lymphoid structures in high-grade serous tubo-ovarian carcinoma: anatomical site matters

**DOI:** 10.1007/s00262-024-03911-2

**Published:** 2025-01-03

**Authors:** Sofia Westbom-Fremer, Lena Tran, Anna Ebbesson, Laura Martin de la Fuente, Jenny-Maria Jönsson, Päivi Kannisto, Srinivas Veerla, Ingrid Hedenfalk

**Affiliations:** 1 Division of Oncology, Department of Clinical Sciences Lund, and Lund University Cancer Center, Lund University, Lund, Sweden; 2 Division of Obstetrics and Gynaecology, Department of Clinical Sciences, Skåne University Hospital, Lund University, Lund, Sweden

**Keywords:** Tertiary lymphoid structure, High-grade serous carcinoma, Metastasis, Prognosis

## Abstract

Tertiary lymphoid structures (TLS) in the tumor microenvironment are prognostically beneficial in many solid cancer types. Reports on TLS in high-grade serous tubo-ovarian carcinoma (HGSC) are few, and the prognostic impact is unclear. We investigated mature TLS (mTLS), immature TLS (iTLS) and lymphoid aggregates (LA) in primary adnexal tumors (PTs) and synchronous omental/peritoneal metastases (pMets) of HGSC. Whole H&E slides were scrutinized for mTLS and LA in a population-based cohort of 130 cases with stage III-IV HGSC. The immune cell tumor infiltration was evaluated with single chromogenic immunohistochemistry (IHC) on a tissue microarray (TMA) from the same cases. Selected whole slides (PT *n* = 11, pMet *n* = 10) of the cases most abundant in mTLS and LA were further investigated with multiplex IHC and immunofluorescence using digital image analysis (QuPath), to confirm TLS status and map the T and B lymphocyte subtypes. The results showed that mTLS were more common in pMets than in PTs but did not have an independent prognostic impact on overall or progression-free survival. The presence of mTLS correlated with intratumoral infiltration of CD8^+^ cytotoxic T cells, FOXP3^+^ regulatory T cells and PD-1^+^ lymphocytes in pMets only. Although overall mTLS cell composition was similar between PTs and pMets, the outer zones of mTLS in PTs were more immune cell-rich. In conclusion, our results indicate differences in TLS presence and cellular elements between primary adnexal tumors and synchronous peritoneal metastases, which are important to consider when conducting studies of the immune environment in HGSC.

**Supplementary Information:**

The online version contains supplementary material available at 10.1007/s00262-024-03911-2.

## Introduction

High-grade serous tubo-ovarian carcinoma (HGSC) usually presents as advanced-stage disease with metastases to the omentum and peritoneal surfaces. Its typical molecular features include homologous repair deficiency (HRD), *TP53* mutations and chromosomal instability, resulting in somatic copy number alterations and substantial tumor heterogeneity [1–3]. Many HGSCs display rich tumor infiltration of CD8^+^ effector T lymphocytes, which is prognostically beneficial [4, 5], but treatment with immune checkpoint inhibitors (ICI) has shown limited efficacy in this entity. The intratumoral T cells express inhibitory receptors resulting in impaired effector functions coupled to ICI resistance [6, 7].

Some reports on tertiary lymphoid structures (TLS) suggest that they correlate with better survival in HGSC [8–10], whereas others do not conclude a prognostic benefit from TLS presence [11, 12]. Cases with TLS formation have higher numbers of CD8^+^ and CD4^+^ tumor infiltrating lymphocytes (TIL) as well as plasma cells [8, 11, 12]. Kroeger et al. report a trend of TLS being more common in omental metastases than primary tumors in an investigation limited to 30 cases [8]. A single-cell RNA sequencing and multiplex immunofluorescence (mIF) mapping of different HGSC lesions has shown that immune cell densities vary between anatomical sites within patients, with higher lymphocyte and CD8^+^ TIL fractions observed in metastatic sites compared to the adnexae, and enrichment of dysfunctional T cells in adnexal sites [6].

The peritoneum and omentum harbor specific immunologically active tissues which filter the peritoneal fluid. The interplay between mesothelial cells, specialized fibroblastic stromal cells and tissue-resident and recruited immune cells is seemingly unique but insufficiently understood at these sites. The numerous vascularized and lymphocyte-rich omental milky spots do not contain follicular dendritic cells (FDCs). Hence, the milky spots lack the properties of mature TLS which contain germinal centers where antigen presentation and B and T cell maturation can take place [13, 14]. Parallels of the omental and peritoneal immune modeling functions have not been described in the female adnexae and the ovaries in particular contain very few immune cells [15, 16].

As several solid cancer types which are rich in TLS show favorable responses to immune checkpoint inhibition, TLS structures are believed to have important functions in regulating the intratumoral immune environment [17–20]. Presumably, strategies for inducing TLS formation or altering TLS functions have the potential to transform cancer tumors into a more ICI-sensitive state. In this study, our aim was to map the presence and composition of TLS and lymphoid aggregates (LA), and their association with the intratumoral immune infiltrates in primary tumors (PTs) compared to synchronous omental and peritoneal metastases (pMets). We investigated a tissue microarray (TMA) and whole slide images with morphologic methods, including single and multiplex immunohistochemistry (IHC) and multiplex immunofluorescence (mIF), in a consecutive cohort of 130 cases of advanced HGSC.

## Material and methods

### Study subjects and specimens

Ethical approval was granted by the Ethics Committee at Lund University, Sweden (EPN 2014/717), waiving the requirement for informed consent. Consecutive cases diagnosed with HGSC (*n* = 156) at the Department of Gynecology and Obstetrics in the southern Swedish healthcare region between 2011 and 2015 were included as previously described [21]. All tumor tissue was chemotherapy-naïve and evaluated according to the WHO 2020 classification by a gynecologic pathologist (SWF) and staged according to International Federation of Gynecology and Obstetrics (FIGO) criteria [22, 23]. Only patients with stage III or IV disease were included in the analyses due to the small number of cases diagnosed in stage I and II. A total of 26 cases were excluded due to histology other than HGSC (*n* = 11), insufficient material (*n* = 4) or FIGO stage I or II (*n* = 11), rendering 130 included cases. The Gynecologic Cancer InterGroup definition of platinum sensitivity (Vancouver June 2010) was used to classify progression-free intervals (PFI) [24]. If tested, *BRCA1/2* mutation status attained in the clinical setting was recorded. HRD status for a limited number of cases analyzed with OncoScan SNP arrays in a previous study was available [25].

The TMA included an average of six cores per patient from PTs and pMets, when available. Cores were punched from tumor cell-rich areas devoid of necrosis or technical artifacts, without taking immune infiltration into consideration.

### Identification of mature TLS and lymphoid aggregates

Hematoxylin and eosin (H&E) stained whole slides from blocks with tissue donated to the TMA, two blocks from PTs and one block from pMets, were examined to identify TLS and lymphoid aggregates (LA) within one millimeter or less from tumor epithelial cells. Only mature TLS (mTLS), i.e., collections of lymphocytes with a concentric round architecture and visible germinal centers (GC) with a surrounding darker zone of lymphocytes, were considered true TLS on H&E sections. Lymphoid aggregates were defined as collections of 100 or more lymphocytes tightly arranged without other intervening cell types. Immature TLS (iTLS), which lack proper germinal centers but contain inner B cells and outer T cell zones, could not be distinguished from LA and could therefore not be evaluated on H&E slides.

### Immunohistochemistry

Sections, 4-µm thick, from the TMA blocks were stained according to details in Supplementary Table 1. The TMA sections were mounted on charged glass slides and baked at 60 °C for 60 min. Standard control tissues used in the clinical setting were added to all slides. DAB was used for detection for all markers, with the addition of Discovery Teal (Ventana Medical Systems Inc, Tucson, AZ) for the dual IHC staining. The sections were stained on the Ventana Benchmark Ultra, Ventana Discovery Ultra (Ventana) or Dako Autostainer (Agilent Technologies, Solna, Sweden) platforms depending on whether the protocols were set up in the clinical or research facilities. The dual IHC slides were scanned with NanoZoomer S60 (Hamamatsu, Japan).

### Multiplex immunofluorescence

Whole slides from the PTs (*n* = 11) and pMets (*n* = 10) most abundant in mTLS and LA were further analyzed with multiplex immunofluorescence (mIF) using the OPAL system from Akoya Biosciences (USA). Three different panels were developed to characterize the lymphocyte populations in TLS and LA: a T cell panel (including markers CD20, CD8, PD-1, FOXP3, TCF7 and CK7), a B cell maturation panel (CD79a, AICDA, MUM1, BCL6, KI67 and CK7), and a B cell activation panel (CD79a, CD4, CD40L, CD40 and CK7). Details on antibody clones and vendors are given in Supplementary Table 2.

In short, 3 µm sections from the FFPE blocks were mounted on TOMO IHC adhesive slides (Histolab Products, Askim, Sweden) and baked vertically at 60 °C for 60 min. All stainings were performed on the fully automated Discovery Ultra instrument. After antigen retrieval, the slides for mIF were incubated with the primary antibody of the first cycle followed by horseradish peroxidase-conjugated secondary antibody (760–4311 or 760–4310; Ventana). Tyramide signal amplification opal fluorophores (Akoya Biosciences, USA) were used for detection. The procedure was sequentially repeated for each marker with a denaturation step after each cycle. Lastly, the slides were counterstained with spectral DAPI (Akoya Biosciences, USA) and mounted with Prolong Diamond Antifade Mountant (Thermo Fisher Scientific, USA). The slides were scanned with the PhenoImager HT system and spectral unmixing was performed in the scanning procedure (Akoya Biosciences, USA).

### Scoring of TMA slides

All stains were scored by a gynecopathologist (SWF) and some of them by a second scorer: CD68, CD3 and PD-1 by LMF (previously reported in [21]) and Cyclin E1 and D1 by JMJ. Cases with discrepant results were re-assessed to reach consensus. Scorers were blinded from clinical data. PD-L1 expression was evaluated in tumor-associated macrophages (TAM) and tumor infiltrating lymphocytes (TIL) separately (previously reported in [21]). Tumor intraepithelial immune cell infiltration for all markers was assessed by determining the fraction of cells expressing the marker in the whole core (number of marker expressing immune cells in tumor epithelial compartment)/(total number of tumor cells + total number of immune cells within tumor epithelial compartment), with the exclusion of areas with acute inflammation and necrosis. The scoring categories 0%, < 1%, 1%, 2–4% and ≥ 5% were used. Cyclin E1, D1 and c-Myc were scored in five percent intervals of positive tumor cell nuclei in all cores combined for each patient and site, and the level of intensity (weak, moderate, strong).

### Multiplex immunofluorescence image analysis

Digital image analysis on whole slide mIF was performed in the open-source software QuPath version 0.5.0 [26]. Using the B cell markers CD20 and CD79a in the mIF images, alongside consecutive sections with dual IHC of CD79a/CD23, mTLS, immature TLS (iTLS) and LA were annotated in the mIF images. As we could not find any validated definitions for differentiation between mTLS, iTLS and LA across different publications, the following criteria based on the H&E and CD79a/CD23 images were set up before the TLS/LA classification was conducted. For a structure to be classed as an mTLS, there had to be a minimum of 100 CD79a positive B cells and a core network of at least ten CD23 positive FDCs forming a concentric rounded structure corresponding to a germinal center. Structures with more than 100 B cells and less than 10 FDCs forming no concentric network were labeled iTLS and those without any FDCs were categorized as LA. Regions of interest (ROI) were annotated, and each mTLS was divided into three zones: FDC-containing germinal center (GC), B cell-rich inner zone (mTLS_IZ) and a peripheral outer zone (mTLS_OZ). As the mTLS outer borders were not clearly delimited, the mTLS_OZ was standardized and annotated as a 100-μm broad rim surrounding the mTLS_IZ, containing mostly T cells. A similar 100 μm wide peripheral zone was also annotated for iTLS and LA. The built-in QuPath cell detection command was used, and a composite object classifier was trained for cell classification. All ROIs were then reviewed to ensure adequate cell classification. Cell interactions were investigated with QuPath centroid distances and visualized with R statistical environment version 4.3.3 and the ggplot2 package [27, 28].

### Statistics

Statistical analyses were performed using the IBM Statistical Package for the Social Sciences (SPSS), version 28. Associations between marker expression and TLS or LA within each anatomical site were assessed with the Pearson χ^2^-statistic and the distributions of markers between PTs and pMets were evaluated with McNemar´s test. Comparisons within the mTLS and LA groups were conducted with Students *t-test* for age, Pearson Chi-square test for FIGO stage, WHO status and residual disease status, Fisher´s exact test for *BRCA*/HRD status, and Mann–Whitney *U* test for PFI. Differences in the mean densities between mIF markers on whole slides were tested with the Mann–Whitney *U* test. As this test is not suited for paired samples, one random slide from each of the three paired samples was excluded from the analysis. The survival endpoints used were 5-year overall survival (OS), defined as the time interval between date of diagnosis and death of any cause, and progression-free survival (PFS), defined as the time interval between date of diagnosis and recorded disease progression or death of any cause. The Kaplan–Meier method was used with the Log-rank test as indicator of statistical differences in survival between groups and the effect of the investigated factor on OS and PFS was estimated using univariable and multivariable cox regression and expressed as hazard ratios (HRs) with 95% confidence intervals. As our investigation was explorative and multiple comparisons were made, *P*-values of < 0.01 were regarded as significant to account for multiplicity. All P-values were two-sided.

## Results

### Clinical characteristics

Of the 130 population-based cases of advanced-stage HGSC, 119 had tissue from PTs and 113 from pMets (*n* = 90 omentum and *n* = 23 peritoneum) included in the TMA. Cases with both PT and pMet tissue counted 104. The median follow-up time was 39 (0–137) months. The clinical characteristics in relation to the presence of TLS and LAs are given in Supplementary Table 3, and details on treatment and rates of complete resections have been previously described [21]. There was no association between the presence of TLS or LA and age, FIGO stage, residual disease after surgery, WHO status, PFI or *BRCA* mutation/homologous repair status.

### Mature TLS in H&E sections

**Fig. 1:**
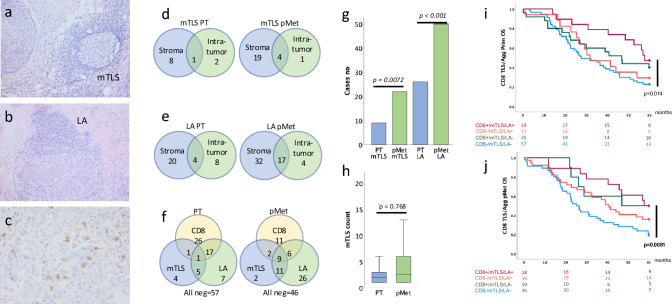
Mature tertiary lymphoid structures and lymphoid aggregates in HGSC primary tumors (PTs) and peritoneal metastases (pMets). Example images of mTLS (**a**, HE × 10), LA (**b**, HE × 10) and CD8high infiltration (**c**, × 40). Distribution of mTLS **d** and LA **e** in different tissue compartments and anatomical sites. Presence of mTLS, LA and CD8high infiltration in primary tumors and peritoneal metastases, with one case missing CD8 score due to core loss **f**. Distribution of cases with mTLS and LA in the different sites (Related samples McNemar´s test, **g**) and total number of mTLS in mTLS positive cases divided by anatomical site (Mann–Whitney U test, **h**). Overall survival stratified by CD8- and mTLS/LA-positivity estimated with Log-rank test in primary tumors **i** and peritoneal metastases **j**

Mature TLS were more common in pMets than in PTs (21 and 9% of cases respectively, *p* = 0.0072) as were LA (47 and 27% respectively, *p* < 0.001). Example images and tissue compartment distribution are shown in Fig. 1. Most mTLS and LA were situated in the stromal compartments (73 and 63% respectively in PTs, 79 and 60% respectively in pMets). Of the cases with tissue from both PTs and pMets, five cases (5%) had mTLS and 20 (19%) had LA in both anatomical locations. The presence of mTLS was associated with the presence of LA in pMets but not in PTs (Pearson Chi-square test, *p* = < 0.001 and 0.030). Regarding OS and PFS, no impact of mTLS or LA alone was observed. When grouping cases with mTLS and/or LA together in PTs or pMets, positive cases showed a trend, not reaching statistical significance, toward longer OS but not PFS in pMets (Log rank *p* = 0.043 and 0.34). No differences in OS or PFS were observed for PTs (Log rank *p* = 0.11 and 0.13, Supplementary Table 4).

### Intratumoral immune infiltration

The different inflammatory markers were dichotomized into high and low expression based on the core with the highest expression at each site, creating as equally-sized groups as possible, resulting in cut-offs of ≥ 5% in PD-L1 TAMs, ≥ 2% for CD3 and CD68, ≥ 1% for CD8 and PD-1 and > 0% positive intratumoral cells for FOXP3, CD20, CD138 and PD-L1 TILs. Example images of high expression are shown in Supplementary Fig. 1.

In general, high expression of single inflammation markers associated well or showed trends toward associations, between the different markers within both PTs and pMets separately, except for most markers versus PD-L1 expression (regardless of cell type expressing PD-L1), detailed in Supplementary Table 5. High expression of CD138 and PD-L1 TAM was more common in PTs than pMets (McNemar’s test, *p* = 0.0014 and < 0.001).

### Mature TLS, LA and intratumoral inflammatory infiltrates

The distributions of high and low expression of the single markers and their relation to mTLS and LA in PTs and pMets are summarized in Table 1. None of the inflammatory markers were associated with mTLS in PTs. There was, however, an association between high expression of CD8, CD138 and PD-L1 TILs and LA in PTs (*p* = 0.0053, 0.0026 and 0.0077). Contrary to PTs, pMets with mTLS were more likely to have high CD8^+^, FOXP3^+^ and PD-1^+^ TILs fractions (*p* = 0.0071, 0.0039, 0.0025). CD138^+^ plasma cell infiltration was associated with LA in pMets (*p* = 0.0026).

**Table 1:** Mature tertiary lymphoid structures and lymphoid aggregates in HGSC primary tumors and metastases

		Primary tumors						
		N (%)	mTLS		*P* value*	LA		*P* value*
			+	−		+	−	
			11 (9)	108 (91)	*P*	32 (27)	87 (73)	*P*
mTLS	+					6 (19)	5 (6)	0.030
	−					26 (81)	82 (94)	
CD3	High	60 (50)	4 (36)	56 (52)	0.33	21 (66)	39 (45)	0.044
	Low	59 (50)	7 (64)	52 (48)		11 (34)	48 (55)	
CD8**	High	44 (37)	2 (18)	42 (39)	0.17	18 (58)	26 (30)	**0.0053**
	Low	74 (63)	9 (82)	65 (61)		13 (42)	61 (70)	
FOXP3***	High	100(85)	9 (82)	91 (86)	0.72	28 (90)	72 (84)	0.37
	Low	17 (15)	2 (18)	15 (14)		3 (10)	14 (16)	
PD-1	High	65 (55)	5 (45)	60 (56)	0.52	23 (72)	42 (48)	0.022
	Low	54 (45)	6 (55)	48 (44)		9 (28)	45 (52)	
CD20**	High	52 (44)	6 (60)	46 (43)	0.30	19 (61)	33 (38)	0.024
	Low	66 (56)	4 (40)	62 (57)		12 (39)	54 (62)	
CD138	High	38 (32)	3 (27)	35 (32)	0.73	17 (53)	21 (24)	**0.0026**
	Low	81 (68)	8 (73)	73 (68)		15 (47)	66 (76)	
CD68	High	53 (45)	6 (55)	47 (44)	0.48	19 (59)	34 (39)	0.048
	Low	66 (55)	5 (45)	61 (56)		13 (41)	53 (61)	
PD-L1 TAM	High	53 (45)	7 (64)	46 (43)	0.18	19 (59)	34 (39)	0.048
	Low	66 (55)	4 (36)	62 (57)		13 (41)	53 (61)	
PD-L1 TIL	High	28 (24)	3 (23)	25 (23)	0.76	13 (41)	15 (17)	**0.0077**
	Low	91 (76)	8 (77)	83 (77)		19 (59)	72 (83)	
Cyclin E1	High	73 (61)	9 (82)	64 (59)	0.14	23 (72)	50 (57)	0.15
	Low	46 (39)	2 (18)	44 (41)		9 (28)	37 (43)	
Cyclin D1	High	36 (30)	5 (45)	31 (29)	0.25	11 (34)	25 (29)	0.55
	Low	83 (70)	6 (55)	77 (71)		21 (66)	62 (71)	
C-Myc	High	24 (20)	2 (18)	22 (20)	0.86	7 (22)	17 (20)	0.78
	Low	95 (80)	9 82)	86 (80)		25 (78)	70 (80)	

		Metastases						
		N (%)	mTLS		*P* value	LA		*P* value
			+	−		+	−	
			24 (21)	89 (79)		53 (47)	60 (53)	
mTLS	+	24 (21)				20 (38)	4 (7)	** < 0.001**
	−	89 (79)				33 (62)	56 (93)	
CD3**	High	44 (39)	12 (50)	32 (36)	0.23	25 (48)	19 (32)	0.076
	Low	68 (61)	12 (50)	56 (64)		27 (52)	41 (68)	
CD8	High	28 (25)	11 (46)	17 (19)	**0.0071**	16 (30)	12 (20)	0.21
	Low	85 (75)	13 (54)	72 (81)		37 (70)	48 (80)	
FOXP3**	High	87 (78)	23 (100)	64 (72)	**0.0039**	42 (81)	45 (75)	0.47
	Low	25 (22)	0	25 (28)		10 (19)	15 (25)	
PD-1***	High	51 (46)	17 (74)	34 (39)	**0.0025**	30 (59)	21 (35)	0.012
	Low	60 (54)	6 (26)	54 (61)		21 (41)	39 (65)	
CD20***	High	46 (41)	13 (57)	33 (37)	0.080	26 (50)	19 (32)	0.057
	Low	66 (59)	10 (43)	56 (63)		26 (50)	40 (68)	
CD138**	High	16 (14)	5 (22)	11 (12)	0.25	13 (25)	3 (5)	**0.0026**
	Low	96 (86)	18 (78)	78 (88)		39 (75)	57 (95)	
CD68**	High	43 (38)	14 (58)	28 (32)	0.017	26 (50)	16 (27)	0.011
	Low	70 (62)	10 (42)	60 (68)		26 (50)	44 (73)	
PD-L1 TAM	High	27 (24)	8 (33)	19 (21)	0.22	12 (23)	15 (25)	0.77
	Low	86 (76)	16 (67)	70 (79)		41 (77)	45 (75)	
PD-L1 TIL	High	19 (17)	7 (29)	12 (13)	0.068	9 (17)	10 (17)	0.96
	Low	94 (83)	17 (71)	77 (87)		44 (83)	50 (83)	
Cyclin E1**	High	71 (63)	17 (71)	54 (61)	0.39	39 (74)	32 (54)	0.034
	Low	41 (37)	7 (29)	34 (39)		14 (26)	27 (46)	
Cyclin D1	High	35 (31)	4 (17)	31 (35)	0.088	14 (26)	21 (35)	0.33
	Low	78 (69)	20 (83)	58 (65)		39 (74)	39 (65)	
C-Myc**	High	22 (20)	4 (17)	18 (20)	0.68	11(21)	11 (19)	0.78
	Low	90 (80)	20 (83)	70 (80)		42 (79)	48 (81)	

*Pearson Chi-square test

**One case or ***two cases missing core loss/too few tumor cells in TMA

High CD8 expression in pMets was associated with a longer PFS (*p* = 0.0029), and there was a trend toward prognostic benefit of high CD8 expression for OS and PFS in PTs (Log rank *p* = 0.018 and 0.025) and OS in pMets (Log rank *p* = 0.022 and 0.0029), Supplementary Table 4. The prognostic effect on OS of mTLS and/or LA presence combined with intratumoral CD8 expression is summarized in Kaplan–Meier plots in Fig. 1. CD8high/mTLS/LA + cases in PTs as well as pMets showed trends toward improved OS and PFS compared to CD8low/mTLS/LA- cases (Log rank *p* = 0.014 and 0.015 for PTs, and *p* = 0.0081 and 0.010 for pMets, PFS data not shown). There was no statistically significant impact on survival in CD8high cases without mTLS/LA, but it is noteworthy that these subgroups were comparatively small. Multivariate analysis including the known prognostic factors age, FIGO stage and residual tumor showed trends toward an independent impact of CD8/TLS/LA-positivity on OS and PFS in PTs only (*p* = 0.016, HR 0.44, 95%CI 0.22–0.86 and *p* = 0.035, HR 0.52, 95%CI 0.28–0.96 respectively, Supplementary Table 4). However, an interaction Cox regression model did not support an independent effect of mTLS alone or mTLS/LA on survival outcomes in the CD8high cases at either site.

### TLS composition

**Fig. 2:**
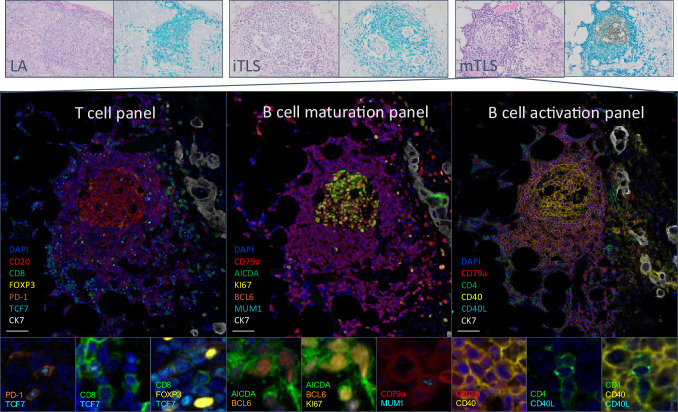
Example microscopic images of lymphoid aggregates (LA), immature (iTLS) and mature tertiary lymphoid structures (mTLS). Top: LA, iTLS and mTLS, H&E and dual CD79a (teal) and CD23 (DAB) IHC, 10x. LA had no CD23^+^ follicular dendritic cells (FDCs), iTLS had less than ten FDCs and mTLS had ten or more FDCs forming a concentric network. Middle: The three multiplexed immunofluorescence panels on an mTLS (20x). Bottom: Example images of different cell phenotypes (40x). The scale bars in the middle pictures equal 50 µm

**Fig. 3:**
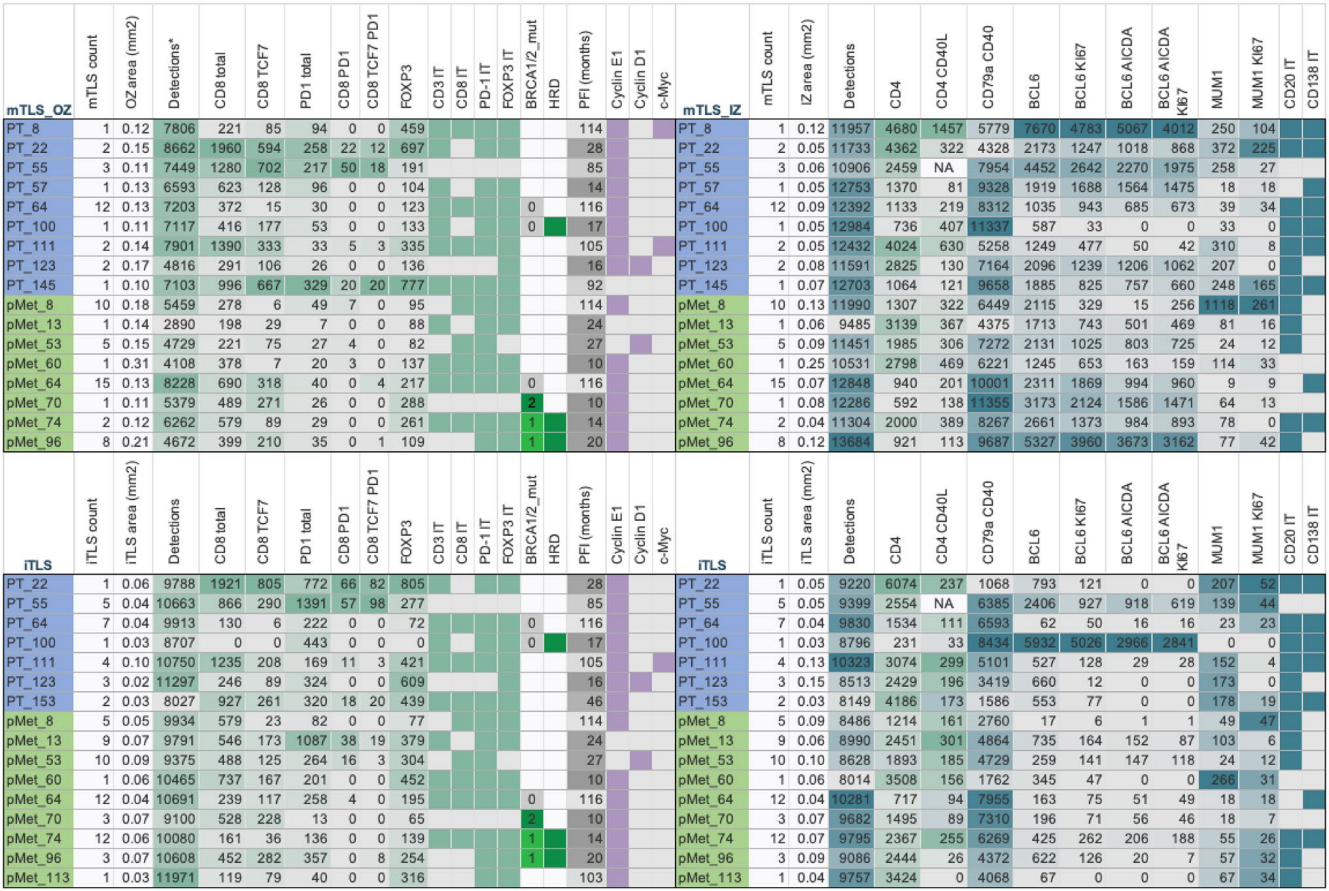
Immune cell densities in mature (mTLS) and immature tertiary lymphoid structures (iTLS). Upper left: Mean mTLS outer zone areas (mm2), T cell densities (cells/mm2) and intratumoral (IT) infiltration of single T cell and cyclinE1/cyclinD1/c-Myc IHC markers. *BRCA* mutation and HRD status are given when known, alongside progression-free interval (PFI, months). Upper right: Mean mTLS inner zone areas, B cell maturation and activation panel densities and IT infiltration of B cell IHC markers. Lower panels: Corresponding variables in iTLS depicting cell densities in iTLS. Single IHC IT immune infiltration and cyclins/c-Myc were dichotomized into high/low. * *p* = 0.0022 for PT vs pMet

Among the slides with the most abundant TLS-like structures, four cases were excluded from the whole-slide analysis due to a missing paraffin block (one PT case with mTLS) and loss of TLS/LA in new H&E sections (two PT cases rich in LA and one pMet with mTLS). Example images of the different structures in whole-slide IHC and mIF are shown in Fig. 2. The total counts of mTLS and iTLS, characterized according to the CD23/CD79a stain, are conveyed in Fig. 3, along with mean cell densities in mTLS_OZ (T cell panel), mTLS_IZ (B cell panels) and iTLS (all panels). The cell contents of the remaining mTLS zones and LA are given in Supplementary Figs. 2 and 3. The overall mean cell densities in mTLS_OZ were higher in PTs than in pMets when assessed in the mIF T cell panel (*p* = 0.0022).

**Fig. 4:**
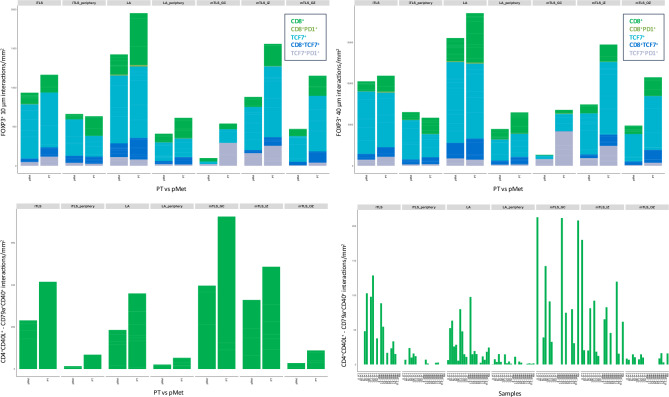
Immune cell interactions within lymphoid aggregates (LA), mature (mTLS) and immature tertiary lymphoid structures (iTLS). Stacked mean FOXP3 interaction densities for cells within centroid-to-centroid distances of up to 10 μm (cells touching, top left) and 40 μm (possible interaction, top right), in iTLS, LA and the separate mTLS zones in PTs and pMets. Stacked interaction densities of B cell activation measured as CD79a^+^CD40^+^ B cell centroid-to-centroid distances of 10 μm from CD4^+^CD40L^+^ helper T cell. B cell activation interactions stratified on the individual samples (bottom right). GC—germinal center, IZ—inner zone, OZ—outer zone

Considering the cell composition of mTLS, iTLS and LA, the structures contained all expected cell types and interactions in both PTs and pMets. The mean cell densities did not differ between sites in the IZ and GC in mTLS (Supplementary Fig. 2). However, examination of the outer T cell-rich zone of mTLS showed higher mean densities of CD8 and PD-1 positive lymphocytes in the PT cases, although this did not reach statistical significance (*p* = 0.040 and 0.029). The B lymphocyte panels showed no statistically significant differences in mean densities between sites for the included cell types. T cell interactions with FOXP3^+^ regulatory T cells at distances of 10 μm (cells touching) and 40 μm (possible interaction), based on centroid-to-centroid distances are depicted in Fig. 4, along with 10 μm B cell activating interactions between CD79a^+^CD40^+^ B cells and CD4^+^CD40L^+^ helper T cells. There was a marked variation in the number of interactions between cases (Fig. 4 for B cell activation and Supplementary Fig. 4 for FOXP3 interactions). The overall trend, although not subjected to statistical testing due to the limited number of samples and marked inter-sample variation, suggested higher interaction densities for FOXP3 as well as B cell CD40-CD40L activation in PTs.

### Molecular profiling

We did not observe any association with mTLS or LA presence in the few cases where *BRCA1/2* mutation status and/or HRD score was known (Supplementary Table 3). High expression of cyclins was determined as > 50% tumor cells with moderate to strong intensity, and for c-Myc > 50% positive tumor cells with any intensity. Cyclin E1 status in PTs and pMets showed no co-variation, and the same was true for cyclin D1 and c-Myc separately, (Supplementary Table 5). There was a negative association between cyclin D1 and PD-1 and PD-L1 TAM in PTs (*p* = 0.0021 and 0.0013), and CD8 and PD-1 in pMets (*p* = 0.0065 and 0.0062). Cyclin E1 and c-Myc expression were not associated with intratumoral immune infiltration. There were trends toward associations between cyclin E1 positivity and LA in pMets (*p* = 0.034) and between mTLS/LA combined and high cyclin E1 expression in pMets (*p* = 0.015) but not in PTs (*p* = 0.080).

## Discussion

Our investigation shows that mTLS are more common in pMets than in PTs in HGSC and were associated with intratumoral infiltration of CD8^+^ effector T cells, PD-1^+^ lymphocytes, FOXP3^+^ regulatory T cells in pMets. There were no such associations in PTs. The initiating mechanisms, constitutions, and functions of TLS may differ depending on the type of tissue or organ where they are situated and the triggering processes behind their development [29–31]. The specialized peritoneal immune milieu could potentially promote, or at least facilitate TLS formation in tissue-specific ways, and the contingent role of the omental milky spots in this process is unclear. The immune cell repertoire and inflammatory responses in the female adnexae, the ovaries in particular, are less well studied. Investigations on limited samples show a sparse presence of resident immune cells in single-cell RNA sequencing series of benign ovaries from gender reassignment and fertility preservation surgeries [15, 16]. Although inflammatory infiltrates can be massive in cases of tubo-ovarian abscess formation or tumor growth, these immune cells are likely recruited rather than resident. The local production of steroid hormones in the ovaries, which persists after menopause albeit with markedly lower activity, also constitutes a factor which presumably entails effects on immune responses in the female adnexae, as estrogen is known to exert a considerable, mostly suppressive, influence on immune and inflammatory processes [32, 33].

Unlike the prognostic benefit from mTLS presence described in many other solid tumors like pulmonary, colorectal, urothelial and breast carcinoma as well as malignant melanoma [17–20, 34], we could not deduce an independent prognostic impact of mTLS and LA in our case cohort. Previous reports show conflicting results as to the prognostic value of TLS in HGSC, which may largely depend on a lack of consensus definitions of what constitutes a TLS [8–11]. The recent elaborate multi-omics characterization of TLS in HGSC by Kasikova et al. sheds more light, as they, like we, found that improved disease outcome likely is attributed to the intratumoral immune cell infiltration rather than to TLS formation [12]. They also deduced a survival benefit for individuals with iTLS only compared to cases with mTLS, which might further explain previous diverse reports on the prognostic impact of TLS in HGSC.

The associations between mTLS and tumor infiltrating CD8^+^, PD-1^+^ and FOXP3^+^ TILs have partly been described previously in HGSC, but our findings suggest an association only in pMets. Kroeger et al. found strong associations between TLS and intratumoral CD8^+^, CD4^+^ and CD20^+^ TILs in 30 cases of mixed primary and metastatic HGSC [8]. Kasikova et al., not specifying HGSC tumor site, also reported higher intratumoral densities of CD8^+^ effector cells, CD3^+^FOXP3^+^ and PD-1^+^ lymphocytes in mTLS positive cases. Further, their results indicate that the scarcity of mTLS in HGSC is associated with an intratumoral CD8^+^ population which is dominated by an ICI-resistant TIM3^+^PD-1^+^ phenotype, in contrast to TLS-rich non-small cell lung cancer, where the opposite is true [12]. In their elaborate characterization of the immune environment in HGSC, Vázquez-Garcia et al. reported lower fractions of lymphocytes and CD8 + T cells and a higher variation of T cell phenotypes and enrichment of dysfunctional T cells in adnexal compared to non-adnexal samples [6]. Our in-depth mapping of the cell composition and cell–cell interactions in PT and pMet TLS and LA revealed overall conformities between the sites and is consistent with descriptions of TLS characterized in other conditions and tissues [30, 31]. However, the outer zone of mTLS in PTs displayed higher total lymphocyte counts and trends toward higher CD8 and PD-1 densities and more FOXP3 to T cell and B cell activating interactions in PT compared to pMet TLS structures. Taken together, these findings indicate potential differences in TLS function and their impact on the intratumoral phenotypes of CD8 effector T cells at the separate anatomical sites.

The lack of information on HRD status for most cases and the variation in eligibility for PARPi treatment, due to study enrollment during a transition time for HRD testing and PARPi treatment in Sweden, pose a weakness in our study. The few cases with known *BRCA*/homologous repair status did not show an association between HRD and TLS/LA presence. This is in line with other studies showing an association between tumor mutation burden, but not *BRCA1/2* mutations, and TLS formation [12]. HRD is coupled to increased activity of the cGAS/STING pathway and STING activation could be a means to induce TLS formation [35, 36]. Based on previous reports of the cGAS/STING pathway being highly expressed and coupled to immune cell infiltration in triple-negative breast cancer with replication stress [37], we explored the expression of cyclin E1, cyclin D1 and c-Myc and immune infiltration. Although not statistically significant, high cyclin E1 protein expression tended to associate with mTLS/LA presence in pMets but not PTs. Notably, the protein expression of cyclin E1 in HGSC does not mirror *CCNE1* amplification in most cases, and we used a comparatively low cut-off for positive expression [38]. Moreover, HGSC with *CCNE1* amplification often have the least immune infiltration among the molecular subtypes and a poor prognosis [39, 40]. Therefore, it seems likely that the TLS/LA positive cases in our cohort harbor other mechanisms behind the high expression of cyclin E1, and the connections between cyclin E1, replicative stress, TLS formation and their plausible relations to cGAS/STING in HGSC remain to be explored in future studies.

In all, our findings suggest differences in the immune environment between PTs and synchronous pMets coupled to TLS in HGSC, which is relevant for understanding the mechanisms of immune evasion and potential therapeutic initiation of tumor-targeted immunity. Our results further stress the importance of considering the anatomical site when mapping and making attempts to remodel the immune landscape of HGSC, especially since the need for therapeutic intervention is more urgent in the setting of recurrent tumor growth, which is most often situated in the serous cavities.

## Supplementary Information

Below is the link to the electronic supplementary material.

Supplementary file1 (PDF 1073 kb)

## Data Availability

The data that support the findings of this study are available from the corresponding author, SWF, upon reasonable request.

## Abbreviations

FDC: Follicular dendritic cell
H&E: Hematoxylin and eosin
HGSC: High-grade serous carcinoma
HRD: Homologous recombination deficiency
ICI: Immune checkpoint inhibitors
IHC: Immunohistochemistry
iTLS: Immature tertiary lymphoid structure
IZ: Inner zone
LA: Lymphoid aggregate
mIF: Multiplex immunofluorescence
mTLS: Mature tertiary lymphoid structure
OS: Overall survival
OZ: Outer zone
PD-L1: Programmed death ligand 1
PD-1: Programmed death protein 1
PFI: Progression-free interval
PFS: Progression-free survival
pMet: Peritoneal metastasis
PT: Primary tumor
TAM: Tumor-associated macrophages
TIL: Tumor-infiltrating lymphocyte
TLS: Tertiary lymphoid structure
TMA: Tissue microarray
