# Supplementary material for: Tertiary lymphoid structures in high-grade serous tubo-ovarian carcinoma: anatomical site matters
Source: Cancer Immunol Immunother. 2025 Jan 3;74(2):56. doi: 10.1007/s00262-024-03911-2 (PMC11699021; doi:10.1007/s00262-024-03911-2)
Supplement: Supplementary file 1 — Supplementary file1 (PDF 1073 kb) [file 262_2024_3911_MOESM1_ESM.pdf]

Supplementary table 1. Single and dual immunohistochemistry protocol conditions.

Antibody	Clone	Vendor	Dilution	Ag retrieval (pH/min/°C)	Platform	AB incubation Min/°C or RT	Control tissue
CD3	Poly	Ventana	1:200	DT K8004 (9/20/98) *	Dako Autostainer	30/RT	Tonsil, appendix
CD8	C8/144B	Dako	1:50	CC1 (8.5/36/95)	Ventana Benchmark Ultra	32/36	Tonsil, appendix
FOXP3	236A/E7	Abcam	1:200	DT K8004 (9/20/98) *	Dako Autostainer	30/RT	Tonsil, appendix
PD-1	NAT105	Cell Marque (Sigma)	1:100	DT 1699 (6/10/120) **	Dako Autostainer	30/RT	Tonsil, appendix
CD20	L26	Ventana	RTU	CC1 (8.5/36/95)	Ventana Benchmark Ultra	32/36	Tonsil, appendix
CD138	B-A38	Ventana	RTU	CC1 (8.5/64/95)	Ventana Benchmark Ultra	48/36	Tonsil, appendix
CD68	PG-M1	Agilent Dako	1:100	DT K8004 (9/20/98) *	Dako Autostainer	30/RT	Tonsil
PD-L1	22C3	Agilent Dako	1:50	DT 1699 (6/10/120) **	Dako Autostainer	30/RT	Placenta, tonsil
Cyclin E1	CCNE1/2460	Abcam	1:100	DT 1699 (6/10/120) **	Dako Autostainer	30/RT	CAMA1 cellpellet***
Cyclin D1	SP4	Ventana	RTU	CC1 (8.5/36/95)	Ventana Benchmark Ultra	36/99	Tonsil
C-Myc	Y69	Abcam	1:50	CC1 (8.5/36/95)	Ventana Benchmark Ultra	32/100	Tonsil
CD79a	SP18	Ventana	RTU	CC1 (8.5/64/95)	Ventana Discovery Ultra	32/37	Tonsil
CD23	1B12	Leica Biosystems	1:25	CC2 (6/8/100)	Ventana Discovery Ultra	32/37	Tonsil

Ag antigen; CC1 Ventana cell conditioning 1; DT Dako Target Retrieval Solution; RT room temperature; RTU ready to use.

* PT-Link

** 2100 Antigen Retriever

*** Antibody specificity was confirmed in CAMA1 wt / CAMA1 Cyclin E1 knockdown FFPE cell pellets

Supplementary table 2. Protocols for the three protocols multiplex immunofluorescence panels.

Antibody	Clone	Vendor	Dilution	Ag retrieval /denaturation (pH/min/°C)	AB incubation Min/°C or RT	Detection	Incubation Dilution/Min/°C
T cell							
PD1	NAT105	Ventana	RTU	CC1 (8.5/64/95)	60/37	Opal 620*	1:100/16/37
CD20	L26	Ventana	1:3 RTU	CC2 (6/8/100)	32/37	Opal 690	1:75/16/37
CD8	C8/144B	Agilent DAKO	1:100	CC2 (6/8/100)	60/37	Opal 520	1:100/16/37
FoxP3	236A-E7	Abcam	1:60	CC2 (6/8/100)	32/37	Opal 570	1:125/16/37
TCF7	C.725.7	Invitrogen	1:60	CC2 (6/8/100)	32/37	Opal 480	1:150/16/37
CK7	SP52	Ventana	RTU	CC2 (6/8/100)	32/37	TSA-DIG	1:300/16/37
						Opal 780	1:25/60/RT
B cell maturation							
AICDA	EPR23436-45	Abcam	1:60	CC1 (8.5/64/95)	32/37	Opal 520	1:100/16/37
MUM1	MUM1p	Agilent DAKO	1:30	CC2 (6/8/100)	60/37	Opal 480	1:400/16/37
BCL6	GI191E/A8	Ventana	RTU	CC2 (6/8/100)	60/37	Opal 620	1:100/16/37
CD79a	SP18	Ventana	1:3 RTU	CC2 (6/8/100)	32/37	Opal 690	1:100/16/37
KI67	MIB-1	Agilent DAKO	1:50	CC2 (6/8/100)	32/27	Opal 570	1:125/16/37
CK7	SP52	Ventana	RTU	CC2 (6/8/100)	32/37	TSA-DIG	1:300/16/37
						Opal 780	1:25/60/RT
B cell activation							
CD4	SP35	Ventana	RTU	CC1 (8.5/48/100)	32/37	Opal 520	1:100/16/37
CD40L	EPR26530-17	Abcam	1:60	CC2 (6/8/100)	60/37	Opal 570	1:125/16/37
CD40	EPR20540	Abcam	1:250	CC2 (6/8/100)	32/37	Opal 480	1:125/16/37
CD79a	SP18	Ventana	1:3 RTU	CC2 (6/8/100)	32/37	Opal 690	1:100/16/37
CK7	SP52	Ventana	RTU	CC2 (6/8/100)	32/37	TSA-DIG	1:300/16/37
						Opal 780	1:25/60/RT

*Antibodies are given in sequence order from top to bottom.
Ag- antigen; CC1- Ventana cell conditioning 1; CC2 – Ventana cell conditioning 2; RT- room temperature; RTU- ready to use.

Supplementary table 3. Mature tertiary lymphoid structures, lymphocyte aggregates and clinical parameters in primary tumors and peritoneal metastases.

		Primary tumors					
		mTLS			LA		
		+	-	P	+	-	P
N (%)	119	11 (9)	108 (91)		32 (27)	87 (73)	
Age							
Mean	66	63	66	0.25[a]	67	65	0.29[a]
Range	43-86	56-68	43-86		52-85	43-86	
FIGO stage							
III	93 (78)	8 (73)	85 (79)	0.65[b]	26 (81)	67 (77)	0.62[b]
IV	26 (22)	3 (27)	23 (21)		6 (19)	20 (23)	
Residual disease							
Y	52 (44)	5 (45)	47 (44)	0.90[b]	13 (41)	39 (45)	0.68[b]
N	67 (56)	6 (55)	61 (56)		19 (59)	48 (55)	
WHO status							
0	108 (91)	7 (64)	88 (81)	0.16[b]	26 (81)	69 (79)	0.82[b]
1-3	11 (9)	4 (36)	20 (19)		6 (19)	18 (21)	
PFI							
>12 months	59 (50)	7 (64)	52 (50)	0.28[c]	16 (52)	43 (51)	0.78[c]
6-12 months	29 (24)	3 (27)	26 (25)		9 (29)	20 (24)	
< 6 months	27 (23)	1 (9)	26 (25)		6 (19)	21 (25)	
No platinum	4 (3)						
BRCA/HRD							
Proficient	7 (25)	0	7 (27)	0.56[d]	2 (29)	5 (24)	0.58[d]
Deficient	21 (75)	2 (100)	19 (73)		5 (71)	16 (76)	
Overall survival				HR (95% CI)[e]			HR (95% CI)[e]
Events/person years	82/380	6/42	76/338	0.61 (0.27-1.41)	21/114	61/266	0.77 (0.47-1.27)
5y OS (%)*	37 (31)	5 (45)	32 (30)		11 (34)	26 (30)	
Progression-free survival							
Events/person years	100/250	8/30	92/220	0.63 (0.31-1.30)	25/73	75/178	0.82 (0.52-1.29)
5y PFS (%)*	19 (16)	3/(27)	16 (15)		7 (22)	12 (14)	
		Peritoneal metastases					
		mTLS			LA		
		+	-	P	+	-	P
N (%)	113	24 (21)	89 (79)		53 (47)	60 (53)	
Age							
Mean	66	65	67	0.58[a]	65	67	0.31[a]
Range	43-86	52-80	43-86		48-86	43-85	
FIGO stage							
III	88 (78)	19 (79)	69 (78)	0.86[b]	42 (79)	46 (77)	0.74[b]
IV	25 (22)	5 (21)	20 (22)		11 (21)	14 (23)	
Residual disease							
Y	54 (48)	9 (38)	45 (51)	0.26[b]	24 (45)	30 (50)	0.62[b]
N	59 (52)	15 (63)	44 (49)		29 (55)	30 (50)	
WHO status							
0	89 (79)	18 (75)	71 (80)	0.61[b]	44 (83)	45 (75)	0.30[b]
1-3	24 (21)	6 (25)	18 (20)		9 (17)	15 (25)	
PFI							
>12 months	54 (48)	13 (59)	41 (48)	0.43[c]	25 (49)	29 (52)	0.97[c]
6-12 months	25 (22)	4 (18)	21 (25)		14 (27)	11 (20)	
< 6 months	28 (25)	5 (23)	23 (27)		12 (24)	16 (29)	
No platinum	6 (5)						
BRCA/HRD							
Proficient	7 (25)	0	7 (32)	0.26[d]	3 (25)	10 (53)	0.60[d]
Deficient	19 (75)	4 (100)	15 (68)		9 (75)	9 (47)	
Site							
Omentum	90 (80)	21 (88)	69 (78)	0.28[b]	45 (85)	45 (75)	0.19[b]
Peritoneum	23 (20)	3 (13)	20 (22)		8 (15)	15 (25)	
Overall survival				HR (95% CI)[e]			HR (95% CI)[e]
Events/person years	76/357	13/82	63/275	0.68 (0.37-1.23)	32/184	44/173	0.67 (0.42-1.05)
5y OS (%)*	37 (33)	11 (54)	26 (29)		21 (40)	16 (27)	
Progression-free survival							
Events/person years	96/226	18/57	78/169	0.71 (0.43-1.20)	44/111	52/115	0.91 (0.61-1.36)
5y PFS (%)	17 (15)	6 (25)	11 (12)		9 (17)	8 (13)	

* Two cases lost to follow up were missing five-year survival data. [a]t test, [b]Pearson Chi-square test, [c]Mann-Whitney U test, [d]Fisher´s exact test, [e]Cox regression analysis, univariate.

Supplementary table 4. Mature TLS, LA and CD8 intratumoral infiltration and relation to overall survival and progression-free survival.

	**Primary tumor**			**Peritoneal metastases**		
	**Overall survival**					
	Log rank	Univariable Cox		Log rank	Univariable Cox	
	p	HR	95 % CI	p	HR	95 % CI
mTLS yes vs no	0.25	0.61	0.27-1.41	0.20	0.68	0.37-1.23
LA yes vs no	0.31	0.77	0.47-1.27	0.078	0.67	0.42-1.05
mTLS/LA yes vs no	0.11	0.68	0.42-1.10	0.043	0.63	0.40-0.99
CD8 high vs low	0.018	0.57	0.35-0.91	0.022	0.52	0.30-0.92
	**Progression-free survival**					
mTLS yes vs no	0.21	0.63	0.31-1.30	0.20	0.72	0.43-1.20
LA yes vs no	0.38	0.82	0.52-1.29	0.65	0.91	0.61-1.36
mTLS/LA yes vs no	0.13	0.71	0.46-1.10	0.34	0.82	0.55-1.23
CD8 high vs low	0.025	0.62	0.40-0.95	**0.0036**	**0.46**	**0.28-0.78**
	**Overall survival**					
	Multivariable Cox			Multivarable Cox		
	p	HR	95% CI	p	HR	95% CI
CD8high/TLS⁺/LA⁺ yes vs no	0.016	0.44	0.22-0.86	0.35	0.70	0.33-1.5
Age >70 years yes vs no	**<0.001**	**3.6**	**2.3-5.9**	**<0.001**	**2.4**	**1.5-4.0**
FIGO Stage 3 vs 4	**<0.001**	**3.7**	**2.2-6.2**	**<0.001**	**2.9**	**1.8-4.7**
WHO status 0 vs 1-3	0.019	0.53	0.32-0.90	0.34	0.76	0.44-1.3
Residual disease no vs yes	**0.0087**	**0.55**	**0.35-0.86**	0.046	0.62	0.39-0.99
	**Progression-free survival**					
	p	HR	95% CI	p	HR	95% CI
CD8high/TLS⁺/LA⁺ yes vs no	0.035	0.52	0.28-0.96	0.39	0.75	0.40-1.4
Age >70 years yes vs no	**<0.001**	**2.2**	**1.4-3.4**	0.015	1.8	1.1-2.8
FIGO Stage 3 vs 4	**<0.001**	**3.5**	**2.1-5.9**	**<0.001**	**3.9**	**2.3-6.5**
WHO status 0 vs 1-3	0.54	0.86	0.53-1.4	0.75	0.92	0.56-1.5
Residual disease no vs yes	**0.0037**	**0.54**	**0.36-0.82**	0.029	0.63	0.42-0.95

	CD8	CD3	FoxP3	PD-1	CD20	CD138	CD68	PD-L1 TAM	PD-L1 TIL	Cyclin E1	Cyclin D1	c-Myc
CD8	0.015	**<0.001**	0.0018	**<0.001**	**0.0079**	**<0.001**	**<0.001**	0.074	**<0.001**	0.63	0.091	0.84
CD3	**<0.001**	0.016	**<0.001**	**<0.001**	**<0.001**	**0.0020**	**<0.001**	0.067	**<0.001**	0.23	0.098	0.19
FOXP3	0.018	**0.0016**	0.049	**<0.001**	**0.0027**	0.049	**<0.001**	**0.0034**	0.11	0.74	0.53	0.66
PD-1	**<0.001**	**<0.001**	**0.0080**	0.041	0.011	0.014	**<0.001**	**<0.001**	**<0.001**	0.67	**0.0021***	0.61
CD20	0.095	0.023	0.044	**0.0033**	0.65	**<0.001**	**0.0081**	0.15	0.17	0.21	0.65	0.18
CD138	**0.0048**	**0.0026**	0.32	**<0.001**	0.41	**0.0014**	0.016	0.12	0.45	0.28	0.83	0.51
CD68	**<0.001**	**<0.001**	**0.0048**	0.30	0.11	0.56	0.052	0.11	**<0.001**	0.19	0.41	0.29
PD-L1 TAM	**<0.001**	**<0.001**	**<0.001**	**<0.001**	0.043	0.39	0.066	**<0.001**	0.15	0.014	**0.0013***	0.89
PD-L1 TIL	**<0.001**	**<0.001**	0.033	**<0.001**	**0.0047**	0.10	**0.0039**	0.010	0.38	0.010	0.10	0.85
Cyclin E1	0.52	0.90	0.81	0.048	0.56	0.24	0.52	0.18	0.68	0.69	0.013	0.90
Cyclin D1	**0.0065***	0.26	0.87	**0.0062***	1.0	0.49	0.73	0.81	0.26	0.015	1.0	0.063
C-Myc	0.94	0.21	0.61	0.12	0.30	0.65	0.91	0.37	0.069	0.54	0.33	1.0

Primary tumors

Peritoneal metastases

Supplementary table 5. Correlations between single immunohistochemistry markers of inflammation, cyclins and c-Myc within and between anatomical sites. Correlation between high expression of single IHC markers for immune cell infiltration in primary tumors (PT, blue) and omental or peritoneal metastases (pMet, green), Pearson´s Chi square test. White boxes show comparison between immune cell infiltration between the two sites, Related samples McNemar test. * Negative association.

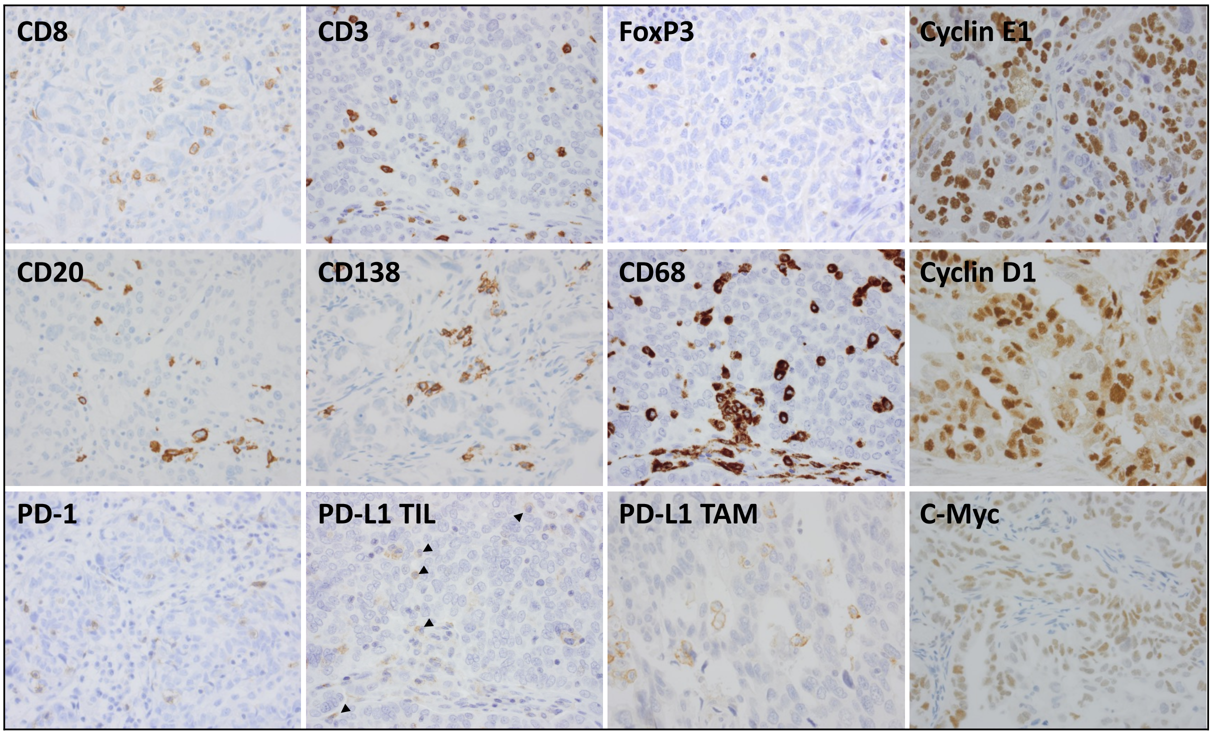

Supplementary figure 1. Example images of intratumor high expression of single immunohistochemistry markers. Black arrowheads indicate PD-1+ lymphocytes.

mTLS GC	mTLS count	GC area (mm2)	Detections	CD8 total	CD8 TCF7	PD1 total	CD8 PD1	CD8 TCF7 PD1	FOXP3	CD3 IT	CD8 IT	PD-1 IT	FOXP3 IT	BRCA1/2_mut	HRD	PFI (months)	Cyclin E1	Cyclin D1	c-Myc
PT_8	1	0.03	18885	0	0	5860	0	0	486							114			
PT_22	2	0.02	19329	1143	40	2711	81	0	321							28			
PT_55	3	0.02	18537	415	176	4194	0	68	479							85			
PT_57	1	0.02	20787	156	0	1611	0	0	104							14			
PT_64	12	0.03	17859	0	0	1514	0	0	66					0		116			
PT_100	1	0.01	23440	0	0	15741	0	0	275					0		17			
PT_111	2	0.01	17270	469	173	1099	0	0	358							105			
PT_123	2	0.02	19416	244	48	1951	0	0	487							16			
PT_145	1	0.01	21897	135	0	1752	0	0	135							92			
pMet_8	10	0.04	17729	53	0	2719	0	0	10							114			
pMet_13	1	0.01	13513	0	0	1134	0	0	0							24			
pMet_53	5	0.02	18495	5	0	3890	0	0	65							27			
pMet_60	1	0.07	18035	269	60	3165	0	0	209							10			
pMet_64	15	0.03	18582	20	0	2989	0	0	241					0		116			
pMet_70	1	0.01	14768	0	0	3408	0	0	0					2		10			
pMet_74	2	0.01	17223	0	0	980	0	0	0					1		14			
pMet_96	8	0.05	19226	18	7	3329	0	0	79					1		20			

mTLS_GC	mTLS count	GC area (mm2)	Detections	CD4	CD4 CD40L	CD79a CD40	BCL6	BCL6 KI67	BCL6 AICDA	BCL6 AICDA KI67	MUM1	MUM1 KI67	CD20 IT	CD138 IT
PT_8	1	0.02	22179	8679	4307	9386	19058	10121	10252	7361	745	482		
PT_22	2	0.01	18376	5181	542	8324	7086	4612	4043	3514	418	260		
PT_55	3	0.01	17937	3732	NA	13765	12212	8820	8583	7597	208	42		
PT_57	1	0.02	17711	1161	116	13472	10328	9236	8640	8143	0	0		
PT_64	12	0.04	16243	1397	506	10349	2052	1875	1288	1261	16	10		
PT_100	1	0.01	20717	1801	1720	17851	7014	2445	1094	708	0	0		
PT_111	2	0.01	19364	4853	1080	7288	4748	2790	358	298	429	0		
PT_123	2	0.02	20570	2080	246	16996	12714	9842	9477	8396	0	0		
PT_145	1	0.01	24598	142	142	23747	7986	4551	4723	4122	601	343		
pMet_8	10	0.05	16965	888	566	12495	686	489	306	690	102	91		
pMet_13	1	0.01	17096	3271	297	10704	7308	3703	2728	2533	195	0		
pMet_53	5	0.02	19042	2099	1215	14809	8672	4690	3819	3568	14	9		
pMet_60	1	0.03	16926	2626	1582	12845	5910	3310	860	838	172	86		
pMet_64	15	0.02	18339	864	458	15495	6542	5289	2740	2652	3	3		
pMet_70	1	0.02	15371	611	407	14302	9803	7015	5441	5082	180	0		
pMet_74	2	0.01	15798	1896	693	12096	8085	4361	3210	2901	79	0		
pMet_96	8	0.04	19490	921	224	14848	14514	11084	10837	9319	77	51		

mTLS IZ	mTLS count	IZ area (mm2)	Detections	CD8 total	CD8 TCF7	PD1 total	CD8 PD1	CD8 TCF7 PD1	FOXP3	CD3 IT	CD8 IT	PD-1 IT	FOXP3 IT	BRCA1/2_mut	HRD	PFI (months)	Cyclin E1	Cyclin D1	c-Myc
PT_8	1	0.14	12543	72	43	2914	0	0	626							114			
PT_22	2	0.10	12165	1806	310	1089	36	12	598							28			
PT_55	3	0.05	13219	950	392	1629	28	99	381							85			
PT_57	1	0.06	12590	663	108	694	0	0	62							14			
PT_64	12	0.09	14092	81	3	597	0	0	52					0		116			
PT_100	1	0.04	16114	94	24	6111	0	0	165					0		17			
PT_111	2	0.07	12579	1263	345	249	4	0	499							105			
PT_123	2	0.11	10742	250	132	402	0	0	795							16			
PT_145	1	0.08	12322	997	689	665	0	25	517							92			
pMet_8	10	0.14	14845	540	28	1135	2	0	91							114			
pMet_13	1	0.06	9385	505	196	1189	0	16	554							24			
pMet_53	5	0.07	12922	132	67	1092	2	0	182							27			
pMet_60	1	0.26	11474	884	137	933	4	0	364							10			
pMet_64	15	0.09	15233	160	48	1214	0	0	236					0		116			
pMet_70	1	0.06	11061	116	50	712	0	0	265					2		10			
pMet_74	2	0.05	11957	46	0	599	0	0	93					1		14			
pMet_96	8	0.20	15316	532	321	1478	2	0	260					1		20			

mTLS_OZ	mTLS count	OZ area (mm2)	Detections	CD4	CD4 CD40L	CD79a CD40	BCL6	BCL6 KI67	BCL6 AICDA	BCL6 AICDA KI67	MUM1	MUM1 KI67	CD20 IT	CD138 IT
PT_8	1	0.09	9123	6521	239	693	607	51	0	0	204	45		
PT_22	2	0.11	9148	6172	307	978	727	107	0	0	627	179		
PT_55	3	0.13	6736	3162	NA	1644	1095	160	150	14	322	29		
PT_57	1	0.11	6230	2281	65	2011	40	16	0	0	56	32		
PT_64	12	0.16	8570	3130	86	1814	17	11	2	2	73	62		
PT_100	1	0.12	6994	648	8	3434	93	9	0	0	0	0		
PT_111	2	0.13	8888	5246	557	1119	717	52	0	0	477	0		
PT_123	2	0.14	4847	1560	87	1404	625	29	3	0	258	10		
PT_145	1	0.13	6033	2115	67	2018	887	121	0	0	383	181		
pMet_8	10	0.16	4790	847	47	481	1836	1304	826	1	113	109		
pMet_13	1	0.13	2839	667	31	124	131	0	0	0	66	8		
pMet_53	5	0.13	2809	915	19	364	30	0	0	0	16	1		
pMet_60	1	0.28	4651	1506	81	188	357	39	0	0	364	35		
pMet_64	15	0.12	8052	2683	26	2032	111	21	0	0	48	41		
pMet_70	1	0.14	6560	2958	21	2612	213	7	0	0	29	0		
pMet_74	2	0.11	6485	3450	145	1676	494	41	0	0	186	47		
pMet_96	8	0.15	5664	1649	27	1217	267	56	5	2	108	46		

Supplementary figure 2. Immune cell densities in mature tertiary lymphoid centers. Top: Mean mTLS germinal center (GC) areas (mm2), T cell densities (cells/mm2) and case intratumor (IT) infiltration of single T cell and cyclinE1/cyclinD1/c-Myc IHC markers (left). B cell panel and IT infiltration of single B cell markers in mature TLS GC (right). *BRCA* mutation and HRD status are given when known, alongside progression-free interval (PFI, months). Lower panel: Corresponding markers in mTLS inner zones (IZ, left) and mTLS outer zones (OZ, right). Single IHC IT immune infiltration and cyclins/c-Myc were dichotomized into high/low.

LA	LA count	LA area (mm2)	Detections*	CD8 total	CD8 TCF7	PD1 total	CD8 PD1	CD8 TCF7 PD1	FOXP3	CD3 IT	CD8 IT	PD-1 IT	FOXP3 IT	BRCA1/2_mut	HRD	PFI (months)	Cyclin E1	Cyclin D1	c-Myc
PT_8	28	0.16	7541	516	159	145	7	2	364							114			
PT_22	14	0.13	9458	2351	154	175	21	5	490							28			
PT_27	2	0.06	10188	1459	720	30	0	16	382							29			
PT_55	15	0.08	9520	1160	677	573	24	37	389							85			
PT_57	16	0.07	8174	1055	184	154	3	4	148							14			
PT_64	8	0.04	10243	172	25	15	0	0	171					0		116			
PT_100	4	0.14	9982	956	331	76	0	2	416					0		17			
PT_111	7	0.05	10051	1915	186	59	10	0	309							105			
PT_123	5	0.04	9883	713	302	151	0	0	524							16			
PT_145	14	0.34	9287	1753	1070	316	19	34	641							92			
PT_153	10	0.08	8317	1695	190	382	84	10	564							46			
pMet_8	24	0.17	8583	874	84	92	3	0	179							114			
pMet_13	5	0.04	8732	979	275	382	53	12	364							24			
pMet_53	12	0.07	8250	718	282	151	22	4	260							27			
pMet_55	6	0.04	8956	901	418	1143	53	77	409							85			
pMet_60	8	0.12	8412	872	269	159	7	4	531							10			
pMet_64	14	0.04	10061	437	178	270	0	0	227					0		116			
pMet_70	15	0.11	7884	422	115	20	0	0	202					2		10			
pMet_74	12	0.12	8441	549	103	59	4	0	162					1		14			
pMet_96	25	0.12	9972	1612	931	214	5	7	488					1		20			
pMet_113	19	0.10	8948	825	233	36	1	0	317							103			

LA	LA count	LA area (mm2)	Detections	CD4	CD4 CD40L	CD79a CD40	BCL6	BCL6 KI67	BCL6 AICDA	BCL6 AICDA KI67	MUM1	MUM1 KI67	CD20 IT	CD138 IT
PT_8	28	0.19	6937	2284	102	874	75	14	1	10	219	71		
PT_22	14	0.18	8873	3615	312	2416	1617	91	6	3	1271	220		
PT_27	2	0.06	8279	2542	241	3830	77	0	8	0	49	17		
PT_55	15	0.11	9514	2387	NA	6418	1485	302	173	61	227	46		
PT_57	16	0.07	7227	2433	133	2593	446	76	2	0	203	115		
PT_64	8	0.04	8626	1809	66	3990	13	11	2	0	12	8		
PT_100	4	0.16	9139	1701	55	5371	326	54	0	0	6	3		
PT_111	7	0.05	9937	4161	478	3274	829	59	0	0	515	4		
PT_123	5	0.10	8049	1923	162	3522	780	46	0	0	292	13		
PT_145	14	0.30	8714	3192	191	3985	781	182	29	14	409	268		
PT_153	10	0.08	7360	2765	112	1277	1882	192	7	3	836	55		
pMet_8	24	0.20	7727	1227	166	2069	215	82	23	21	122	99		
pMet_13	5	0.05	9785	3092	410	4585	699	55	9	0	159	4		
pMet_53	12	0.08	7557	2757	103	2881	159	8	0	0	29	12		
pMet_55	6	0.03	8415	2149	NA	5058	1100	239	4	4	351	155		
pMet_60	8	0.11	7562	3298	253	2431	564	122	9	5	396	81		
pMet_64	14	0.05	8804	2085	77	4067	251	165	130	119	29	29		
pMet_70	15	0.12	8343	1419	46	5676	330	37	3	3	99	11		
pMet_74	12	0.11	7934	2216	116	3145	1379	196	4	9	708	99		
pMet_96	25	0.14	8551	3410	47	2623	1333	374	30	19	579	231		
pMet_113	19	0.09	8720	4249	160	2751	144	23	0	0	46	39		

Supplementary figure 3. Immune cell densities in lymphoid aggregates (LA). Left: Mean LA areas (mm2), T cell densities (cells/mm2) and case intratumor (IT) infiltration of single T cell and cyclinE1/cyclinD1/c-Myc IHC markers. Right: B cell panel and IT infiltration of single B cell markers. *BRCA* mutation and HRD status are given when known, alongside progression-free interval (PFI, months). Single IHC IT immune infiltration and cyclins/c-Myc were dichotomized into high/low. * p=0.043

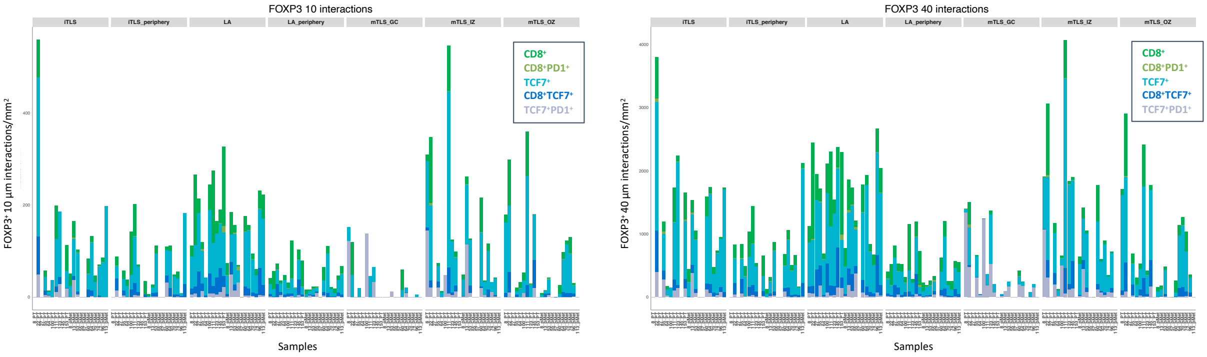

Supplementary figure 4. Stacked FOXP3 centroid-to-centroid distance distances 10 µm (touching) and 40 µm (potential interaction) as measured with interaction density (interactions/mm^2) within in each case/anatomical site and stratified by the different lymphoid structures/mTLS zones in each sample.
